# Protective effects of dexpanthenol against diatrizoate-induced contrast nephropathy via the SIRT1–p53–Bcl-2/Bax pathway in rats

**DOI:** 10.1007/s00210-026-05520-5

**Published:** 2026-06-08

**Authors:** Cenk Mustafa Havabulut, Demet Gündüz, Emine Sarman, İlter İlhan, Muhammet Yusuf Tepebaşı, Abdurrahman Gülal, Mehtap Savran, Halil Aşcı

**Affiliations:** 1 Department of Radiology, Faculty of Medicine, Suleyman Demirel University, Isparta, Turkey; 2 Department of Radiology, Faculty of Medicine, Akdeniz University, Antalya, Turkey; 3 Department of Histology and Embryology, Faculty of Medicine, Afyonkarahisar Health Sciences University, Afyonkarahisar, Turkey; 4 Department of Biochemistry, Faculty of Medicine, Suleyman Demirel University, Isparta, Turkey; 5 Department of Genetics, Faculty of Medicine, Suleyman Demirel University, Isparta, Turkey; 6 Graduate School Of Health Sciences, Suleyman Demirel University, Isparta, Turkey; 7 Department of Pharmacology, Faculty of Medicine, Suleyman Demirel University, Isparta, Turkey

**Keywords:** Apoptosis, Contrast nephropathy, Dexpanthenol, Diatrizoate, Oxidative stress

## Abstract

Contrast agents are widely used in medical imaging to improve tissue differentiation, but they can cause serious inflammatory responses, including contrast-induced nephropathy (CIN). Dexpanthenol (DEX) is known for its antioxidant, anti-inflammatory, and anti-apoptotic properties. This study aimed to investigate the potential protective effects of dexpanthenol in a rat model of diatrizoate-induced CIN. In this study, 32 Wistar albino rats were randomly divided into four groups: control, Urografin (URO; 10 mL/kg, intraperitoneal [i.p.]), URO + DEX (500 mg/kg, i.p. for 3 days), and DEX alone. Renal function markers (serum urea and creatinine), total oxidant status (TOS), total antioxidant status (TAS), and oxidative stress index (OSI) were measured. Histopathological evaluation and immunohistochemical analysis of tumor necrosis factor-alpha (TNF-α) and caspase-3 (Cas-3) were performed. Additionally, SIRT1, Bcl-2, Bax, and p53 mRNA expression levels were assessed. URO administration increased TOS and OSI values and caused significant renal histopathological damage. TNF-α and Cas-3 immunoreactivity, along with Bax and p53 gene expression, were significantly elevated, while Bcl-2 and SIRT1 expression was suppressed. DEX treatment provided partial improvement in these changes, contributed to the preservation of renal architecture, and was associated with improvements in biochemical and molecular parameters approaching those of the control levels. In conclusion, DEX may exert nephroprotective effects against CIN by reducing oxidative stress, inhibiting pro-inflammatory and apoptotic pathways, and increasing anti-apoptotic gene expression. These findings provide a preclinical basis supporting the idea that dexpanthenol is a promising candidate for further translational and clinical studies in contrast-induced renal injury.

## Introductıon

Contrast agents are essential components of modern medical imaging, enhancing tissue contrast and diagnostic accuracy. However, although the ideal contrast agent should have no adverse effects, none of the contrast agents currently in use are completely risk-free and may cause various adverse reactions (Pomara 2015). Among the adverse effects associated with contrast agents, contrast-induced nephropathy (CIN) is one of the most clinically significant complications and has been extensively described in both clinical and experimental studies typically arising after intravenous or intra-arterial administration of contrast media (Kusirisin et al. 2020; Lightfoot et al. 2009; Pucelikova et al. 2008).

In clinical practice, CIN is defined as an increase in serum creatinine of ≥ 25% or ≥ 0.5 mg/dL from baseline within 48–72 h after contrast media exposure (Pucelikova et al. 2008).

Although the detailed pathophysiology of CIN has not been fully clarified, several interacting mechanisms have been identified. Renal medullary hypoxia is considered a central contributor, arising from the imbalance between vasodilator mediators such as nitric oxide and prostaglandins and vasoconstrictors such as adenosine and endothelin (Deray et al. 1991; Heyman et al. 1999). In addition, direct tubular toxicity and excessive production of reactive oxygen species (ROS) have been reported to play essential roles in the initiation and progression of renal damage (Bakris et al. 1990; Katholi et al. 1998). ROS-mediated oxidative stress not only disrupts redox homeostasis but also promotes inflammatory responses through the activation of cytokines, particularly tumor necrosis factor-alpha (TNF-α) (Pucelikova et al. 2008; Romano et al. 2008). Previous studies have demonstrated that iodinated contrast media trigger the release of pro-inflammatory cytokines, particularly TNF-α, which plays a central role in amplifying renal tubular injury and apoptosis (Cho 2022). Furthermore, several studies have reported that iodinated contrast agents induce renal tubular cell apoptosis, demonstrating that apoptotic pathways are a critical component of CIN pathology (Hizoh 2002; Romano et al. 2008).

Within this mechanistic landscape, several apoptosis-related molecular regulators have gained prominence in the evaluation of CIN. Silent mating-type information regulation 2 homolog-1 (SIRT1), a NAD⁺-dependent deacetylase, is known for its cytoprotective and anti-apoptotic properties. In contrast, tumor protein p53 (p53) functions as a key pro-apoptotic transcription factor activated under oxidative and genotoxic stress. The mitochondrial apoptosis-related proteins B-cell lymphoma 2 (Bcl-2) and Bcl-2–associated X protein (Bax) constitute essential determinants of cell survival, with Bcl-2 exerting an anti-apoptotic effect and Bax promoting mitochondrial outer membrane permeabilization and caspase activation. Dysregulation of these pathways, particularly the shift toward increased p53 and Bax expression and decreased SIRT1 and Bcl-2 expression, is a hallmark of contrast-induced tubular injury.

Dexpanthenol (DEX), an alcohol analogue of pantothenic acid and a member of the B-complex vitamin family, has attracted increasing attention due to its cytoprotective biological profile. After metabolic conversion to pantothenic acid, DEX contributes to Coenzyme A synthesis, which plays a central role in cellular energy metabolism, membrane repair and epithelial integrity (Proksch et al. 2017). Additionally, DEX is widely used in dermatological preparations because of its strong tissue penetration and local bioavailability, and has been shown to reduce inflammation, enhance fibroblast activity, accelerate wound healing and protect epithelial structures (Biro et al. 2003). These antioxidant, anti-inflammatory and anti-apoptotic properties suggest that DEX may counteract the oxidative and apoptotic mechanisms underlying contrast-induced renal injury.

**Fig. 1:**
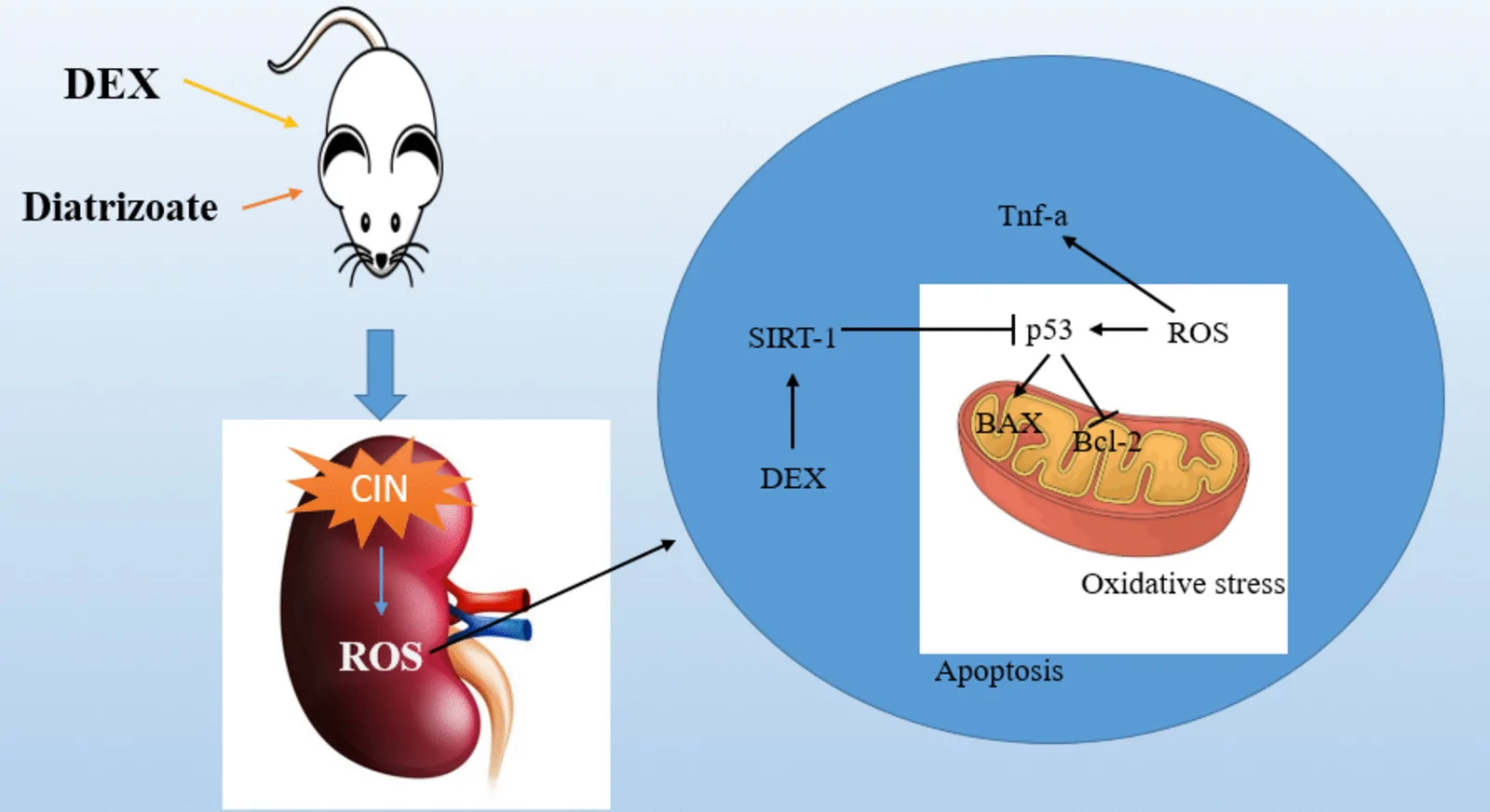
Proposed mechanism of Dexpanthenol (DEX)–mediated protection against diatrizoate-induced contrast-induced nephropathy (CIN). Diatrizoate exposure increases renal oxidative stress (↑ROS), leading to activation of pro-inflammatory (↑TNF-α) and pro-apoptotic pathways (↑p53, ↑BAX) while reducing anti-apoptotic mitochondrial signaling (↓Bcl-2). Excessive ROS production further amplifies mitochondrial dysfunction, resulting in apoptosis of tubular epithelial cells and the development of CIN. Dexpanthenol enhances SIRT1 expression, which suppresses p53-mediated apoptotic signaling, reduces BAX activation, and restores Bcl-2 levels. Through SIRT1–dependent modulation of oxidative stress and apoptosis, DEX attenuates ROS accumulation and exerts a protective effect against CIN in renal tissue

In light of these considerations, the present study aimed to investigate the potential renoprotective effects of dexpanthenol in a rat model of diatrizoate-induced nephrotoxicity. Histopathological, biochemical, immunohistochemical and molecular analyses were performed with particular emphasis on apoptosis-related pathways involving SIRT1, p53, Bcl-2 and Bax to elucidate the mechanisms through which DEX may exert its beneficial effects against contrast-induced renal damage. The inhibitory effect of dexpanthenol on contrast-induced nephropathy at the cellular level is summarized in Fig. 1.

## Materıals and methods

### Animal care

The study was conducted in accordance with the approval of the Local Animal Experiments Ethics Committee (Approval No: 298).

Throughout the study period, the animals were maintained under controlled environmental conditions, including a room temperature of 22 ± 2 °C, relative humidity ranging between 55–60%, and a 12-h light/dark cycle. Standard laboratory chow and drinking water were available ad libitum. All experimental procedures were designed to minimise discomfort and stress. The euthanasia procedure was performed under deep anaesthesia, followed by exsanguination via the inferior vena cava.

### Experimental design

A total of 32 female Wistar albino rats, with a body mass range of 250–350 g, were supplied by the Experimental Animal Research Center of Suleyman Demirel University. Animals were allocated to four experimental groups (n = 8 each) using a computer-generated randomisation procedure.

1. In the control group, rats were given intraperitoneally (i.p.) saline (1 mL/day) for three consecutive days, and an additional 3–4 mL saline dose was administered on day 3.
2. Urograpin (URO) group rats received 1 mL saline i.p for three consecutive days, on day 3, 10 mL/kg of URO (meglumine diatrizoate, 370 mg I/mL) was injected i.p (Yesildağ et al. 2009).
3. URO + DEX group rats received 500 mg/kg DEX i.p. for three consecutive days; on day 3, 10 mL/kg URO was administered i.p (Özden et al. 2023).
4. DEX group rats received 500 mg/kg DEX i.p for three consecutive days; on day 3, 3–4 mL saline was administered i.p

In order to increase the susceptibility to kidney damage prior to contrast administration, a 24-h water restriction was applied (Yenicerioglu et al. 2006**).** At the conclusion of the experimental protocol, the animals were administered deep anaesthesia with 90 mg/kg ketamine and 10 mg/kg xylazine. Following this, surgical exanguination was performed, blood samples were taken, and kidney tissues were removed. The collected samples were then subjected to a comprehensive array of analytical procedures, encompassing histopathological, immunohistochemical, biochemical, and genetic examinations.

### Histopathological and immunohistochemical examination

Renal tissue samples were fixed in 10% neutral buffered formalin, then routinely processed, embedded in paraffin, and sectioned at 5 µm thickness. The prepared sections were deparaffinized and rehydrated using ethanol series of increasing concentrations. Standard hematoxylin–eosin (H&E) staining was performed on the sections for general histopathological evaluation. For immunohistochemical examination, tissue sections were incubated with caspase-3 (Cas-3; FNab01289, Fine Test; 1:100) and tumor necrosis factor-alpha (TNF-α; sc-52746, Santa Cruz; 1:200) antibodies. Visualization of antigen–antibody reactions was performed using a streptavidin–biotin–peroxidase complex-based method (Candan et al. 2024).

All stained sections were analyzed under an Eclipse E-600 Nikon photomicroscope using NIS Elements software (Nikon, Japan). Immunoreactivity was semi-quantitatively scored as follows: 0 (negative), 1 (weak focal), 2 (diffuse moderate) and 3 (strong intense). Scoring was performed using ImageJ 1.46r (NIH, USA) software by a blinded histopathologist.

### Biochemical analysis

Serum biochemical parameters were evaluated by measuring urea and creatinine concentrations using spectrophotometric methods with a Beckman Coulter AU5800 analyzer. Oxidative parameters Total antioxidant status(TAS) and total oxidant status (TOS) were determined with commercial assay kits (Rel Assay Diagnostics, Turkey) based on Erel’s method (Erel 2004).

TAS concentrations were presented as mmol Trolox equivalents/L and TOS concentrations as µmol H₂O₂ equivalents/L. The oxidative stress index (OSI) was calculated using the formula: OSI = (TOS/TAS) × 100. This ratio reflects the global oxidative status in renal tissue (İlhan et al. 2025).

### Reverse transcription-polymerase chain reaction (RT-qPCR)

The collected tissue samples were transferred to pre-labeled, DNase/RNase-free, and pyrogen-free Eppendorf tubes and stored at −80 °C until further processing. On the day of analysis, 1000 µL of GeneAll RiboEx™ solution (Seoul, Korea) was added to each tissue sample. The samples were allowed to reach room temperature and homogenized at low speed using a sonicator. RNA was then isolated. RNA concentration and purity were assessed using a Nanodrop spectrophotometer prior to cDNA synthesis. Complementary DNA (cDNA) synthesis was performed in a thermal cycler according to the manufacturer's protocol provided with the A.B.T.™ cDNA Synthesis Kit (Atlas Biotechnology, Turkey). Primer designs were created by identifying specific mRNA sequences using the NCBI website and testing potential primer sequences (Table 1). Primers were designed, synthesized, lyophilized, and redissolved into a 100 μM stock solution with commercially available, DNase/RNase-free, pyrogen-free, ready-to-use sterile water. These stock solutions were further diluted to a final working concentration of 500 nM for quantitative real-time PCR analysis, in accordance with the manufacturer's recommendations. Quantitative assessment of target gene expression was performed using A.B.T.™ kits (Atlas Biotechnology, Turkey) in conjunction with a Biorad CFX96 real-time PCR system (California, USA). The glyceraldehyde-3-phosphate dehydrogenase (GAPDH) gene was used as an internal reference gene, and Ct values were used for normalization. PCR reactions were prepared in 0.1 mL tubes with a total reaction volume of 20 µL. The reaction mixtures were loaded into the qPCR device and amplified under the thermal cycling conditions specified by the kit manufacturer. All samples were analyzed in triplicate. Ct values were recorded separately for each sample and experimental group. Relative gene expression levels were calculated using the 2^ − ΔΔCt method, and the obtained data were processed for subsequent statistical analysis (Livak and Schmittgen 2001).

**Table 1:** Primer information and gene reference data for quantitative PCR analysis

Gene	Specific Primer sequence(5′–3′) Forward (F)-Reverse (R)	Amplicon size (bp)	Accession number
SIRT1	F: GGTAGTTCCTCGGTGTCCT	152 bp	NM_001414959.1
	R: ACCCAATAACAATGAGGAGGTC		
p53	F: CTCCTCTCCCCAGCAAAAG	151 bp	NM_001429996.1
	R: CCTGCTGTCTCCTGACTCCT		
Bcl-2	F: CATCTCATGCCAAGGGGGAA	284 bp	NM_016993.2
	R: TATCCCACTCGTAGCCCCTC		
BAX	F: CACGTCTGCGGGGAGTCAC	419 bp	NM_017059.2
	R: TAGAAAAGGGCAACCACCCG		
GAPDH (housekeeping)	F: AGTGCCAGCCTCGTCTCATA	248 bp	NM_017008.4
	R: GATGGTGATGGGTTTCCCGT		

SIRT1, silent mating-type information regulator 2 homolog 1; p53, tumor protein p53; Bcl-2, B-cell lymphoma 2; BAX, Bcl-2–associated X protein; GAPDH, glyceraldehyde-3-phosphate dehydrogenase. F, forward primer; R, reverse primer; bp, base pairs.

### Statistical analysis

Statistical analyses were performed using GraphPad Prism version 9.5.1 (GraphPad Software, USA). Data were analyzed using one-way analysis of variance (ANOVA) followed by Tukey’s post hoc test. Variance homogeneity was assessed using Levene’s test prior to ANOVA application. Normality of data distribution was evaluated using the Shapiro–Wilk test. All results are presented as mean ± standard deviation, and a p value < 0.05 was considered statistically significant.

### Histopathological evaluation

Comparative histological analysis showed marked structural differences between the experimental groups in renal tissue. Kidneys from the control group retained normal cortical and medullary organization, with preserved glomerular integrity and intact tubular structures, and no detectable inflammatory response. In contrast, URO administration resulted in extensive tissue damage. Observed abnormalities included reduction in glomerular volume, degeneration of tubular epithelium, interstitial mononuclear inflammatory infiltration, and increased collagen deposition in both cortical and medullary compartments. Nuclear pyknosis and tubular lumen enlargement were also evident in this group (p < 0.001 for all comparisons).

Co-treatment with DEX resulted in significant improvement in some of these pathological findings in URO + DEX group. Compared with the URO group, no significant histological changes were observed in the URO + DEX group in terms of glomerular atrophy, pyknotic nuclear condensation, and atrophic tubules (p > 0.05). However mononuclear infiltration (p < 0.001), collagen deposition (p < 0.001) and collecting tubule dilatation (p < 0.01) were statistically significant. No significant histological changes were observed in the DEX group compared with the control group (p > 0.05 for all parameters).

**Fig. 2:**
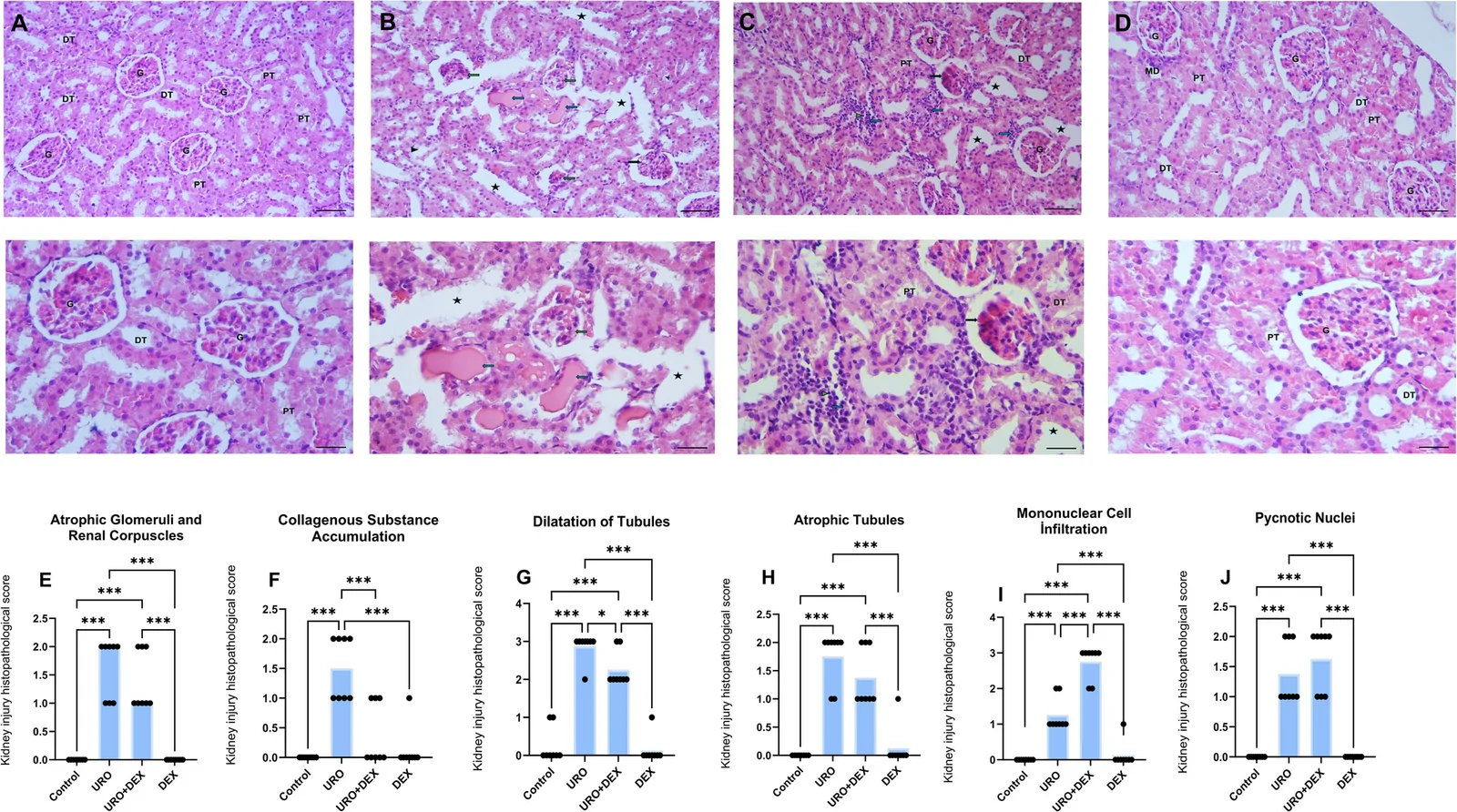
Representative histopathological findings and quantitative scores of renal tissues. (**A**) Normal renal histology observed in the control group, showing intact glomeruli (G), proximal tubules (PT), and distal tubules (DT). (**B**) Severe pathological alterations in the urograpin (URO) group. Normal-appearing glomerulus and renal corpuscle indicated by black arrow; atrophic glomeruli and renal corpuscles marked by green arrow; collagenous material deposition shown by blue arrow; tubular dilatation indicated by black star; atrophic tubules marked with black arrowhead. (**C**) Attenuated histopathological damage in the URO + DEX group, with fewer degenerative changes. (**D**) Nearly normal renal morphology in the DEX group, comparable to the control. (**E-J**) Histopathological scores of graphics. Statistical significance: ***p < 0.001, **p < 0.01, *p < 0.05. Hematoxylin and eosin (H&E) staining; scale bar = 50 μm

Semi-quantitative scoring confirmed these observations; the URO group showed significantly higher injury scores than some other groups, while DEX treatment significantly reduced these scores in some groups, demonstrating a potential protective effect against urographin-induced nephrotoxicity in some groups (Fig. 2).

### TNF-α immunohistochemistry

Immunohistochemical analysis was performed to evaluate TNF-α expression as an indicator of inflammatory activity in renal tissue. In the control group, TNF-α immunostaining was absent or scarcely detectable, reflecting baseline inflammatory conditions. Conversely, the URO group exhibited markedly increased and diffuse TNF-α immunoreactivity in both glomerular structures and tubular epithelial cells, indicating pronounced pro-inflammatory involvement (p < 0.001 for all comparisons).

**Fig. 3:**
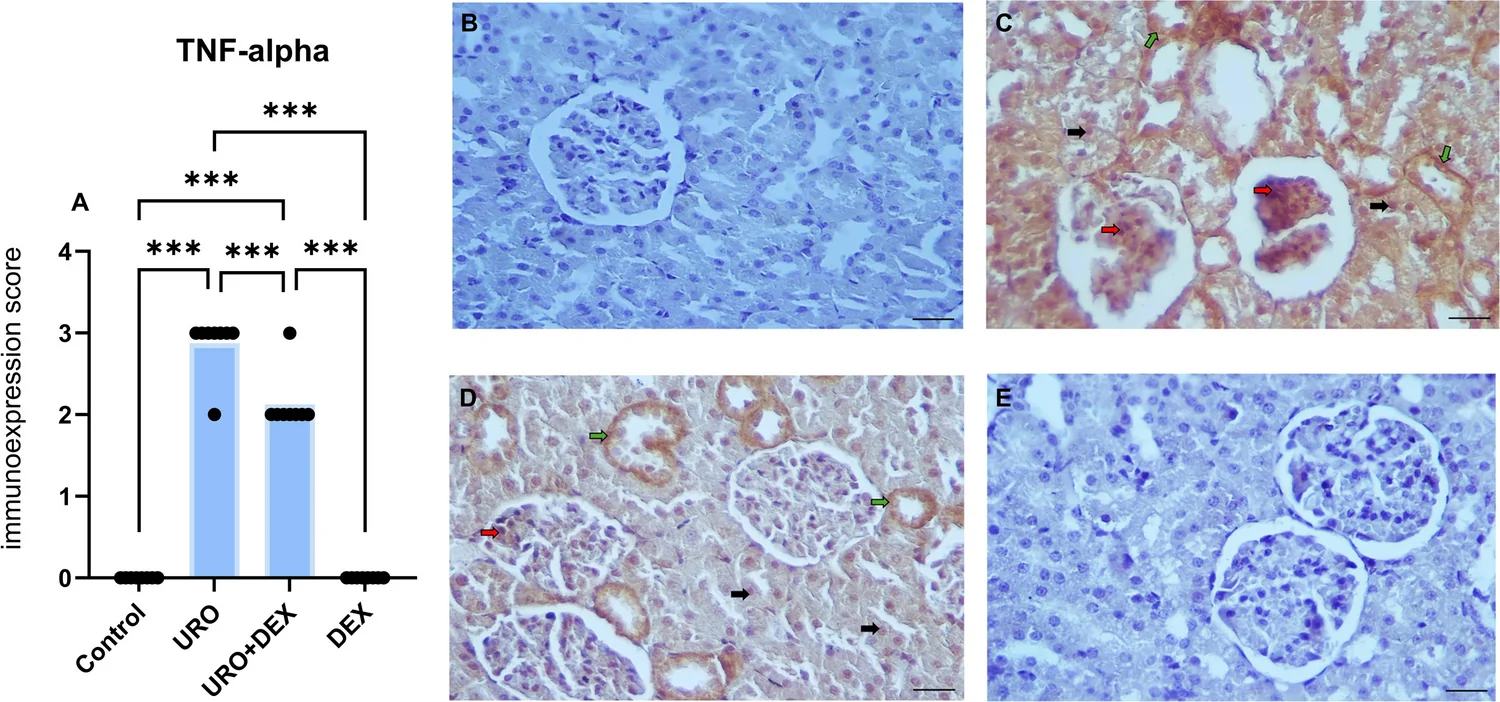
TNF- alpha immunoexpression in renal tissues of experimental groups. (**A**) TNF-α immunoexpression score graphic (**B**) Negative or weak TNF-α expression in the control group. (**C**) Strong positive TNF-α expression in the URO group, with prominent staining in glomeruli (red arrow), tubular epithelium (black arrow), and tubular basement membrane (green arrow). (**D**) Moderate TNF-α immunoreactivity in the URO + DEX group, showing noticeably reduced staining intensity compared to the URO group. (**E**) Nearly absent TNF-α expression in the DEX group. The bar graph represents the semi-quantitative analysis of TNF-α expression levels, scored on a scale of 0–3. Statistical significance: ***p < 0.001, **p < 0.01, *p < 0.05

Co-administration of DEX with URO led to a significant attenuation of TNF-α expression compared with the URO group; however, expression levels remained significantly higher than those observed in the control group (p < 0.001 for both). In contrast, TNF-α expression in the DEX-only group did not differ significantly from control values (p > 0.05) (Fig. 3).

### Cas-3 immunohistochemistry

**Fig. 4:**
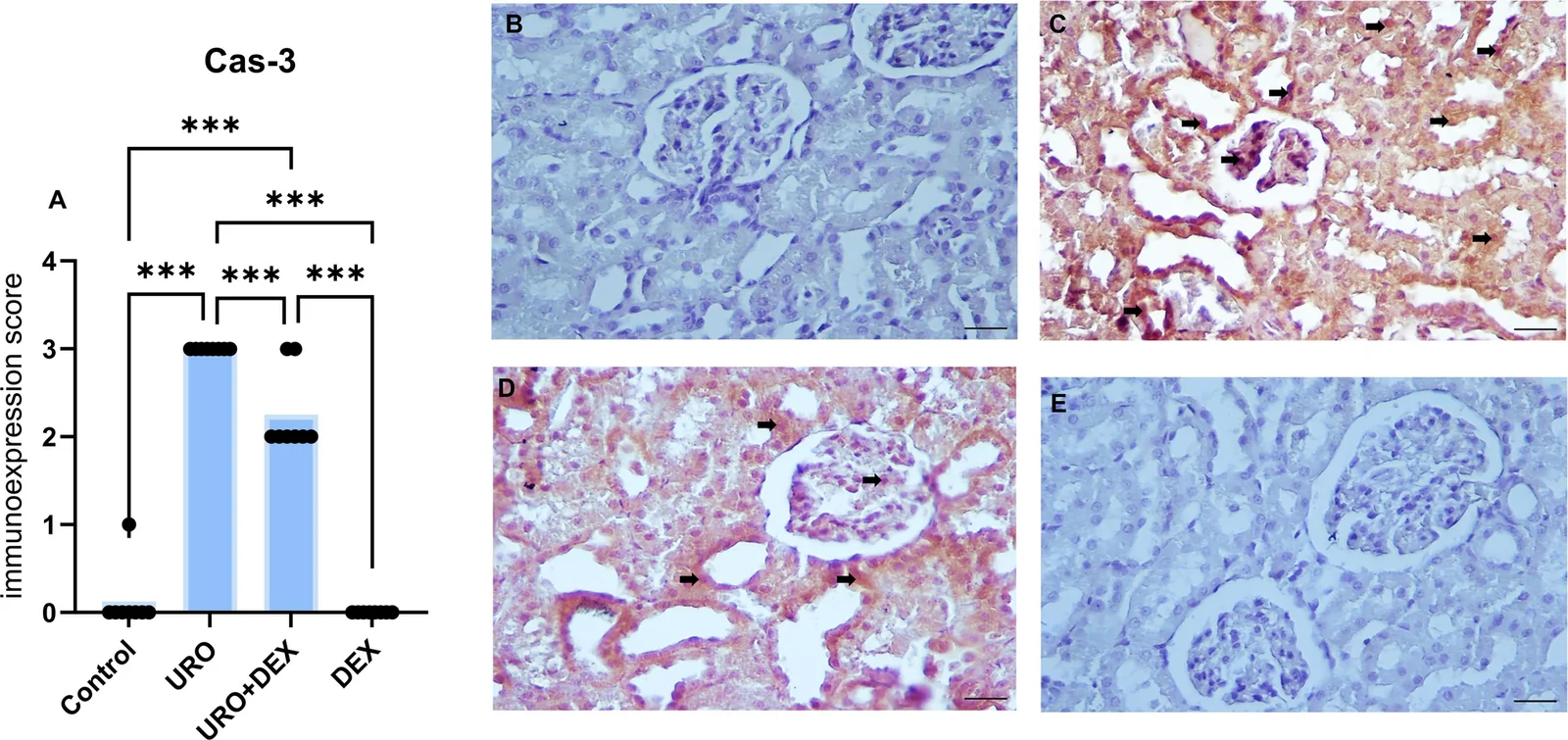
Cas-3 immunoexpression in renal tissues across experimental groups. (**A**) Cas-3 immunoexpression score graphic (**B**) Minimal Cas-3 staining observed in the control group. (**C**) Marked Cas-3 immunoreactivity in the URO group, with strong cytoplasmic expression in tubular epithelial cells (black arrows). (**D**) Moderate Cas-3 expression in the URO + DEX group, showing visibly reduced staining compared to the URO group. (**E**) Cas-3 expression in the DEX group was comparable to that of the control group. The bar graph represents semi-quantitative scoring of Cas-3 immunopositivity. Statistical significance: ***p < 0.001, **p < 0.01, *p < 0.05

Immunohistochemical analysis for caspase-3 (Cas-3) was performed to evaluate apoptotic activity in renal tissues. In the control group, Cas-3 immunoreactivity was minimal, reflecting low basal apoptotic activity under physiological conditions. In contrast, in the URO group, marked Cas-3 positivity was observed, particularly in the cytoplasm of renal tubular epithelial cells, indicating increased apoptotic cell death (p < 0.001).DEX administration significantly reduced Cas-3 expression compared to the URO group (p < 0.001). Although Cas-3 levels in the URO + DEX group were still high compared to the control group, they were significantly lower than in the URO-only group (p < 0.001). No statistically significant difference in Cas-3 expression was found between the DEX and control groups (p > 0.05) (Fig. 4).

### Biochemical results

#### Oxidative Stress Parameters

**Fig. 5:**
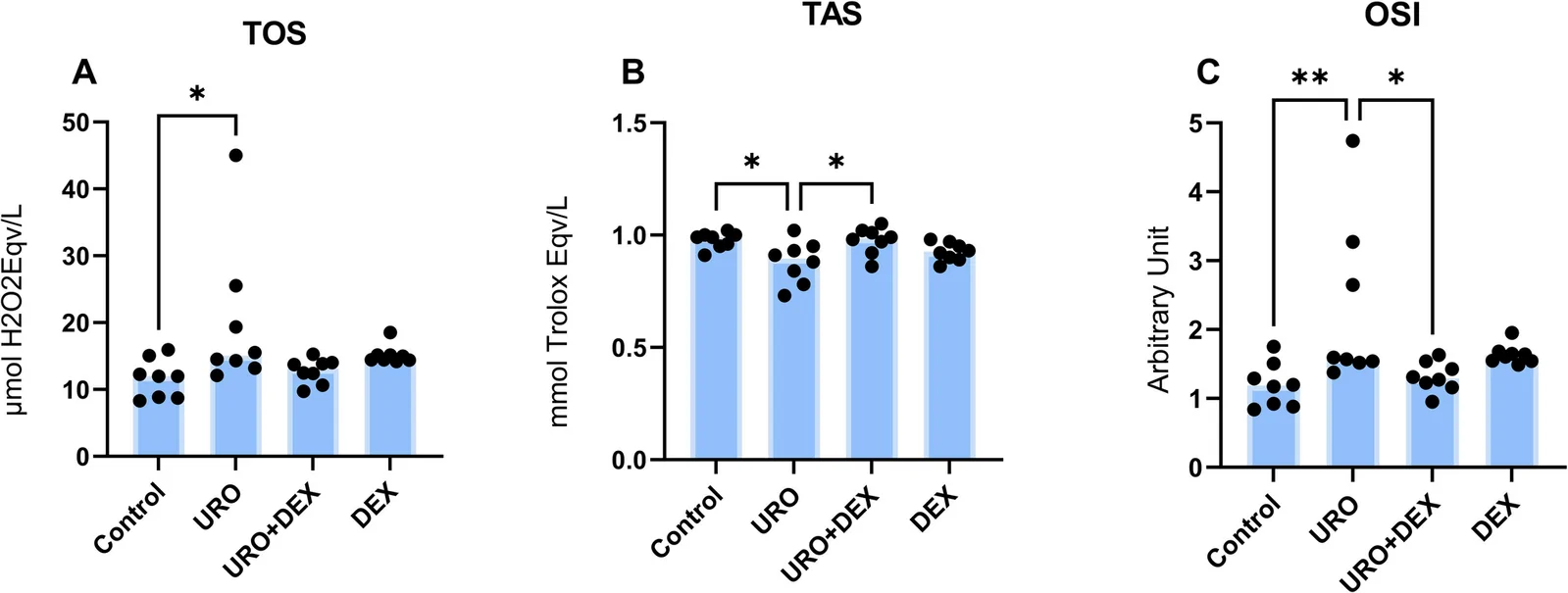
Evaluation of oxidative stress parameters in renal tissues of experimental groups. Bar graphs represent the levels of TOS (**A**), TAS (**B**), and OSI (**C**) in control, URO, URO + DEX, and DEX (Dexpanthenol) groups. ***: p < 0.001, **: p < 0.01, *: p < 0.05

In the URO group, TOS levels were higher compared to the control group (p < 0.05). URO + DEX and DEX groups did not show any significant difference from the control group (p > 0.05). In terms of TAS, a decrease was observed in the URO group compared to the control group (p < 0.05). DEX treatment normalized TAS levels (p < 0.05) and the DEX group remained similar to the control group (p > 0.05). OSI was increased in the URO group compared to the control group (p < 0.01). DEX treatment decreased OSI levels compared to the URO group (p < 0.05). The DEX group was not different from the control group (p > 0.05) (Fig. 5).

#### Serum renal function parameters

**Fig. 6:**
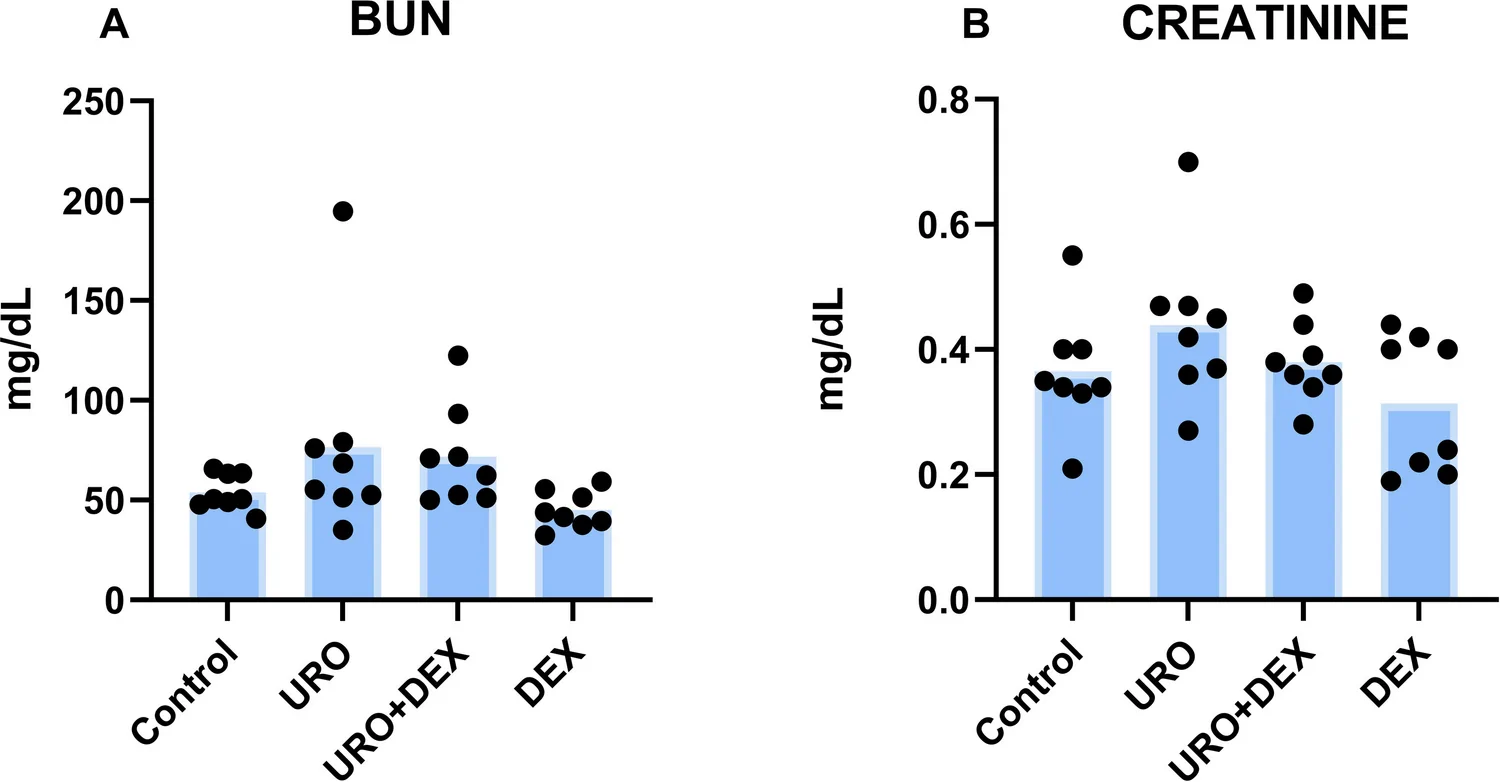
Evaluation of serum renal function parameters in blood. Bar graphs represent the levels of BUN(**A**) and Creatinine(**B**) in control, URO, URO + DEX, and DEX groups. ***: p < 0.001, **: p < 0.01, *: p < 0.05

Serum levels of urea and creatinine were measured to evaluate renal function and detect biochemical alterations secondary to CIN. A comparison of the urea and creatinine levels revealed no statistically significant differences between the groups (Fig. 6).

### qRT-PCR evaluation of apoptosis-related genes (Bcl-2, Bax, SIRT1, p53)

Expression of genes associated with mitochondrial stress-induced apoptosis (Bcl-2, Bax, p53 and SIRT1) was assessed using quantitative real-time PCR. In the URO group, Bcl-2 and SIRT1 mRNA levels were significantly decreased compared to the control group (p < 0.001 for both), while Bax and p53 were significantly increased, indicating a shift toward proapoptotic signaling (p < 0.001 for both).

**Fig. 7:**
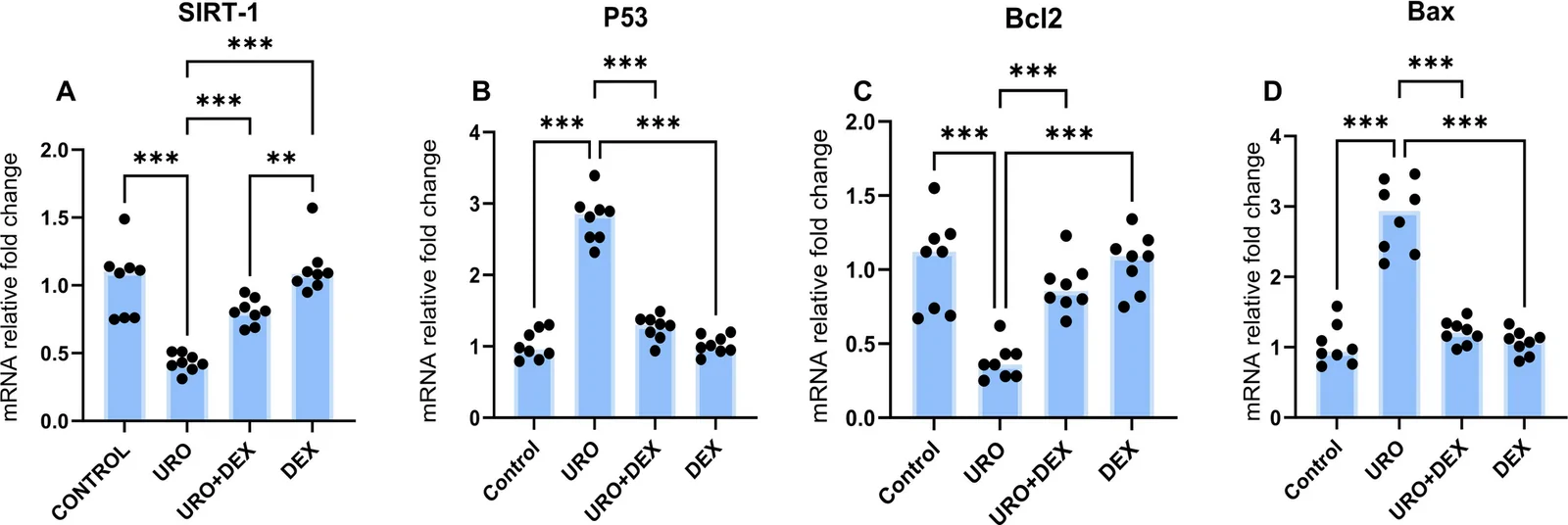
mRNA expression levels of mitochondrial stress-related genes in renal tissues. Bar graphs display the relative fold changes of SIRT1 (**A**), P53 (B), Bcl-2 (**C**) and Bax (**D**) gene expression levels in the control, URO, URO + DEX, and DEX groups. ***: p < 0.001, **: p < 0.01, *: p < 0.05

DEX treatment significantly altered these gene expressions. In the URO + DEX group, Bcl-2 and SIRT1 levels increased compared to the URO group. Bax and p53 expressions were significantly decreased compared to URO group (p < 0.001 for all). There was no significant difference in Bcl-2, Bax, SIRT1 or p53 expression between the DEX alone and control groups (p > 0.05 for all) (Fig. 7).

## Discussıon

CIN remains one of the most frequent causes of hospital-acquired acute kidney injury, and its multifactorial pathophysiology involves medullary hypoxia, oxidative stress, tubular cytotoxicity, endothelial dysfunction, inflammation, and apoptosis. The present study demonstrated that diatrizoate-induced renal injury leads to substantial structural, biochemical, and molecular deterioration, whereas DEX treatment attenuated these deleterious effects. By integrating histopathologic, immunohistochemical, biochemical, and gene expression findings, our study reveals that DEX may exert a renoprotective effect by modulating the SIRT1–p53 axis, restoring mitochondrial homeostasis, and suppressing apoptotic cascades. These results highlight multiple interconnected mechanisms that collectively mitigate contrast-mediated nephrotoxicity.

CIN pathology is driven in part by medullary vasoconstriction and reduced oxygen delivery. Low oxygen tension in the outer medulla augments the generation of ROS, which damages lipids, proteins, and mitochondrial DNA, leading to tubular dysfunction and apoptosis. Previous studies demonstrated that contrast agents shift renal redox balance toward oxidant dominance, resulting in increased OSI and impaired antioxidant capacity (Kusirisin et al. 2020). Consistent with this mechanism, the URO group in this study revealed significantly increased TOS and OSI values, alongside diminished TAS levels. By contrast, DEX successfully reversed these alterations and normalized oxidative parameters, highlighting its antioxidant activity. This effect is consistent with molecular evidence that DEX enhances glutathione synthesis, suppresses lipid peroxidation, and stabilizes cellular membranes under oxidative challenge (Kuru et al. 2023).

Oxidative stress is a key upstream trigger of mitochondrial injury in CIN. Excessive ROS oxidizes cardiolipin and disrupts mitochondrial membrane potential (MOMP), promoting cytochrome-c release and intrinsic pathway activation. The pronounced upregulation of p53 and Bax in the URO group corroborates ROS-mediated DNA damage and mitochondrial apoptotic activation. p53, a sensor of genomic instability, transcriptionally induces Bax and promotes mitochondrial outer membrane permeabilization, a hallmark of irreversible apoptosis. Simultaneously, the downregulation of Bcl-2, an antiapoptotic protein that stabilizes mitochondrial membranes, exacerbates this shift toward apoptosis. Our gene expression findings (Bax↑, p53↑, Bcl-2↓, SIRT1↓) strongly confirm that contrast media activate intrinsic apoptotic pathways in tubular cells, consistent with Romano et al.’s observations of contrast-triggered apoptotic signaling (Romano et al. 2008).

A particularly important mechanistic observation in this study is the significant suppression of SIRT1 in the URO group. SIRT1, a NAD⁺-dependent deacetylase, protects renal cells by deacetylating and inactivating p53, enhancing mitochondrial biogenesis, and promoting anti-inflammatory responses. Reduced SIRT1 activity is known to exacerbate p53 activation, ROS production, and tubular apoptosis. CIN-associated SIRT1 deficiency has been suggested in limited studies, but the present findings provide important molecular confirmation. Importantly, DEX administration restored SIRT1 expression, an effect that may represent one of the central mechanisms underlying its renoprotective potential. Upregulation of SIRT1 likely contributed to the observed reduction in p53 and Bax expression, confirming the restoration of mitochondrial integrity and apoptotic resistance.

The Bcl-2/Bax ratio serves as one of the most reliable indicators of susceptibility to mitochondrial apoptosis. A decreased ratio reflects proapoptotic dominance, cytochrome-c leakage, and Cas-3 activation. In this study, URO reduced Bcl-2 while increasing Bax expression, forming a strong proapoptotic shift. DEX reversed this shift and significantly restored Bcl-2/Bax balance, consistent with a protective mitochondrial phenotype. This molecular improvement was supported by immunohistochemical findings demonstrating that DEX reduced Cas-3 immunopositivity in renal epithelial cells. Together, these results suggest that DEX may help attenuate MOMP-associated mitochondrial apoptotic signaling, suppress caspase activation, and preserve tubular cell viability.

Histopathological analysis supported the biochemical and molecular findings. Diatrizoate caused extensive glomerular atrophy, tubular dilatation, mononuclear infiltration, and collagen accumulation. These classical CIN lesions reflect combined oxidative, apoptotic, and inflammatory injury. DEX ameliorated these structural abnormalities, preserving glomerular architecture, reducing tubular degeneration, and decreasing interstitial inflammation. Our findings are consistent with the histopathological features of contrast-induced nephropathy described by Kiss and Hamar (2016), who reported similar histological deterioration in contrast models, but importantly show that DEX achieves broader protection across multiple renal compartments (Kiss and Hamar 2016). An apparently higher or persistent mononuclear cell infiltration in the DEX-treated injury group should be interpreted cautiously. Mononuclear inflammatory cells may participate not only in tissue injury but also in debris clearance, resolution of inflammation, and remodeling during the repair phase. Therefore, when considered together with the marked reductions in TNF-α and Cas-3 immunoreactivity, normalization of oxidative stress indices, restoration of SIRT1 and Bcl-2 expression, and suppression of p53/Bax signaling, this cellular response more likely represents a residual reparative inflammatory process rather than evidence of aggravated nephrotoxicity.

The immunohistochemical results for Cas-3 and TNF-α matched the gene-level observations, suggesting a tightly coordinated response between inflammatory and apoptotic markers. Elevated TNF-α can enhance mitochondrial apoptosis by promoting p53 activation and Bax translocation. The parallel reduction in both TNF-α and Cas-3 in the DEX-treated group suggests that DEX interrupts this feedback loop and prevents propagation of apoptotic signaling.

The strong recovery of SIRT1 expression observed here is particularly noteworthy because SIRT1 is increasingly recognized as a master regulator of renal stress responses. SIRT1 promotes mitochondrial biogenesis via peroxisome proliferator–activated receptor-gamma coactivator 1-alpha activation, reduces oxidative burden by enhancing antioxidant enzymes such as superoxide dismutase 2, and suppresses inflammation by deacetylating RelA/p65. Therefore, the effect of DEX on SIRT1 may represent an important mechanistic finding of this study, suggesting a potential protective pathway not previously emphasized in CIN research.

The synergistic improvement in OSI, gene expression patterns, TNF-α/Cas-3 immunoreactivity, and histopathology collectively indicates that DEX acts through multiple layers of cellular defense. These findings suggest that the effects of DEX may extend beyond simple antioxidant activity, instead revealing a multifaceted mode of action that includes mitochondrial regulation, anti-inflammatory signaling, transcriptional reprogramming, and epithelial regeneration. This distinguishes DEX from many conventional CIN interventions, such as N-acetylcysteine, which primarily function as simple ROS scavengers and have shown inconsistent clinical benefit.

From a translational perspective, DEX appears to be a promising candidate for further investigation due to its favorable safety profile and multi-targeted protective mechanisms. It is inexpensive, safe, readily available, and widely used in dermatology and wound management. Its established human safety profile creates a favorable foundation for repurposing in nephro-protection. Considering the limitations of current CIN prophylaxis strategies, particularly the variable success of hydration protocols and the limited efficacy of pharmacologic agents, DEX may represent a potential therapeutic approach for preventing renal injury in high-risk patients undergoing radiological contrast procedures.

Although serum urea and creatinine are commonly used clinical markers of renal function, they may not accurately reflect early or subclinical kidney injury in short-term experimental contrast-induced nephropathy (CIN) models. In the present study, the absence of significant differences in serum urea and creatinine levels does not exclude the presence of contrast-induced renal injury, as marked histopathological, oxidative stress, inflammatory, and apoptotic alterations were observed following URO administration. These findings suggest that molecular and structural renal damage may occur before measurable functional impairment becomes evident in this experimental model. Therefore, unchanged serum renal function parameters should be interpreted as a limitation of the experimental model rather than evidence of the absence of nephrotoxicity.

This study has several strengths, including the combined assessment of oxidative stress markers, inflammatory mediators, apoptosis-related proteins, and mitochondrial gene regulatory pathways, and their integration with histopathological validation.

This study has several limitations that should be acknowledged. Although intravenous or intra-arterial administration more closely reflects the clinical use of contrast agents, intraperitoneal administration has also been used in previously published experimental contrast-induced nephropathy studies in rats (Yesildağ et al. 2009). In addition, intraperitoneal administration may be considered an acceptable approach in experimental rat studies as it can provide systemic exposure and practical drug delivery under experimental conditions (Al Shoyaib et al. 2020).

The dexpanthenol dose used in the present study was determined according to previously published experimental studies demonstrating the antioxidant and protective effects of dexpanthenol in different rat injury models, including renal injury models (Ceylan et al. 2011; Doğan et al. 2017; Özden et al. 2023). However, further studies are needed to evaluate the optimal therapeutic dosing and clinical applicability of dexpanthenol in contrast-induced nephropathy.

One limitation of the present study is the absence of protein-level analyses for the investigated molecular targets. Although mRNA expression analyses demonstrated consistent alterations in SIRT1, p53, Bcl-2, and Bax expression, additional protein-level validation would further strengthen the mechanistic findings. In the present study, the parallel findings observed in mRNA expression analyses together with immunohistochemical markers of inflammation and apoptosis (TNF-α and Cas-3), oxidative stress parameters, and histopathological evaluations support the biological relevance of the observed molecular alterations. Another limitation of this study is the lack of evaluation of sensitive early renal injury biomarkers such as Neutrophil Gelatinase-Associated Lipocalin and Kidney Injury Molecule-1. These biomarkers may provide additional information regarding early tubular injury and could be included in future experimental studies on contrast-induced nephropathy.

Future studies incorporating protein-level analyses such as western blotting may provide additional mechanistic insight into the protective effects of dexpanthenol in contrast-induced nephropathy.

## Conclusion

Our findings demonstrate that DEX may confer protective effects against diatrizoate-induced renal injury by reducing oxidative stress, suppressing inflammation, and inhibiting mitochondrial apoptosis through modulation of the SIRT1–p53–Bcl-2/Bax axis. These results suggest a possible mechanistic pathway through which DEX mitigates CIN and these findings suggest that DEX may be a promising candidate for future experimental and translational studies investigating nephroprotection strategies in contrast-induced renal injury.

The absence of significant changes in serum creatinine and urea does not exclude early or subclinical renal injury. In experimental models of contrast-induced nephropathy, histopathological and molecular alterations frequently precede measurable functional impairment, particularly in short-term exposure settings. To the best of our knowledge, this study provides experimental evidence that dexpanthenol may confer protection against contrast-induced renal injury through SIRT1-mediated regulation of mitochondrial apoptosis involving the p53/Bcl-2/Bax signaling axis.

## Data Availability

The datasets generated and/or analyzed during the current study are available from the corresponding author upon reasonable request.
